# Computed tomography-based unsupervised clustering identifies clusters associated with progression free survival in clear cell renal cell carcinoma

**DOI:** 10.1186/s40644-025-00958-x

**Published:** 2025-11-24

**Authors:** Jae Hyon Park, Daeun Choi, Chung Lee, Chang Gon Kim, Sangwoo Kim, Minsun Jung, Jongjin Yoon

**Affiliations:** 1 Department of Radiology, Armed Forces Daejeon Hospital, Daejeon, Korea; 2 Department of Radiology and Research Institute of Radiological Science, Severance Hospital, Yonsei University College of Medicine, Seoul, Korea; 3 Department of Pathology, Severance Hospital, Yonsei University College of Medicine, Seoul, Korea; 4 Division of Medical Oncology, Department of Internal Medicine, Yonsei Cancer Center, Yonsei University College of Medicine, Seoul, Korea; 5 Department of Biomedical Systems Informatics and Brain Korea 21 PLUS Project for Medical Science, Yonsei University College of Medicine, Seoul, Korea

**Keywords:** Carcinoma, Renal cell, Cluster analysis, Multidetector computed tomography, Prognosis, Progression-free survival

## Abstract

**Background:**

This study aimed to develop and validate a radiologic clustering model using CT imaging features to stratify clear cell renal cell carcinoma (ccRCC) patients by prognosis and identify key imaging predictors of 5-year progression free survival (PFS).

**Methods:**

This retrospective study included 164 ccRCC patients with multiphase kidney CT and next-generation sequencing (NGS) between September 2003 and October 2024. Qualitative imaging features were extracted, and unsupervised consensus clustering was performed to classify tumors based on radiologic characteristics. A nomogram-based C1 score was derived from features predictive of the high-risk cluster. Model performance was evaluated using C-index and 5-year area under the receiver operating curve (AUC). Genetic alterations and copy number variations (CNVs) were also analyzed for associations with imaging features and survival.

**Results:**

Clustering revealed two distinct radiologic subtypes. Cluster C1 characterized by aggressive behavior such as tumor heterogeneity (*p* = 0.011), exophytic growth pattern (*p* = 0.002), non-smooth margin (*p* = 0.019), and renal sinus extension (*p* = 0.016), and was independently associated with poorer 5-year PFS (*p* = 0.018). The C1 score demonstrated an AUC of 0.992 for predicting cluster C1 in the test-set. Using a cutoff of 0.75, the model achieved 96.3% sensitivity and 96.4% specificity. For predicting 5-year PFS, the C1 score showed moderate performance (AUC 0.65; C-index 0.65), which improved when combined with nodal/distant metastasis and BAP1 mutation status (AUC 0.71; C-index 0.67).

**Conclusions:**

Radiologic clustering using CT features enables non-invasive prognostic stratification of ccRCC. The C1 score derived from this approach may serve as a practical tool to guide surveillance and treatment decisions.

**Trial registration:**

Retrospectively registered.

**Supplementary Information:**

The online version contains supplementary material available at 10.1186/s40644-025-00958-x.

## Background

Clear cell renal cell carcinoma (ccRCC) accounts for approximately 70% of all primary renal neoplasms [1]. Although most patient present with localized disease and undergo surgery, nearly one-third experience recurrence [2], with about 75% of recurrence occurring within the first five years after surgery [3]. This makes identifying patients at high risk of recurrence essential for informed counseling, tailored surveillance, and selection of candidates for adjuvant therapy.

Traditional risk stratification in ccRCC relies on TNM staging [4], and nuclear grade [5], but their prognostic performance is limited. Several integrated models such as the Mayo Clinic Stage, Size, Grade, and Necrosis (SSIGN) score [6], the 2003 Leibovich model [7], and the UCLA Integrated Staging System (UISS) [8] have been developed to improve accuracy, but they remain largely dependent on pathological features. Currently, these tools are endorsed in daily clinical practice by major guidelines, including the American Urologic Association [9], National Comprehensive Cancer Network [10], and European Association of Urology [11], which recommend using nuclear grade, TNM stage, and 2023 Leibovich model and UISS to guide follow-up protocols.

More recently, next-generation sequencing (NGS) has enabled risk stratification based on gene mutations and copy number variations (CNVs) [12–14]. However, ccRCC is characterized by marked intratumoral heterogeneity at both the pathological and genetic levels [15]. In multi-region sequencing study, only about 34% of detected mutations were shared across all tumor regions, and among known ccRCC driver genes, only VHL was consistently mutated [15]. Histologically, ccRCC also exhibits substantial variation in necrosis and grade [16], with higher-grade tumors demonstrating greater chromosomal instability, which likely contributes to genetic heterogeneity [17]. As such, neither pathology nor genomics-based models relying on partial sampling may capture the full tumor landscape.

Computed tomography (CT), a widely used non-invasive imaging modality, is already integrated into routine evaluation of ccRCC. Qualitative imaging features describing tumor morphology and internal characteristics can serve as surrogates for underlying pathologic and even genetic alterations. Few preliminary studies have reported associations between CT features and genetic alterations [18–20], but these were exploratory in nature, and did not evaluate their utility in predicting clinical outcomes such as survival.

In this study, we aimed to develop and validate a novel classifier for ccRCC using unsupervised clustering of imaging features to predict 5-year cause-specific progression free survival (PFS). We further constructed a nomogram based on this classification for clinical applicability and, using a cohort of ccRCC patients who underwent NGS, explored potential associations between imaging features and genetic alterations.

## Methods

### Study patients

**Fig. 1:**
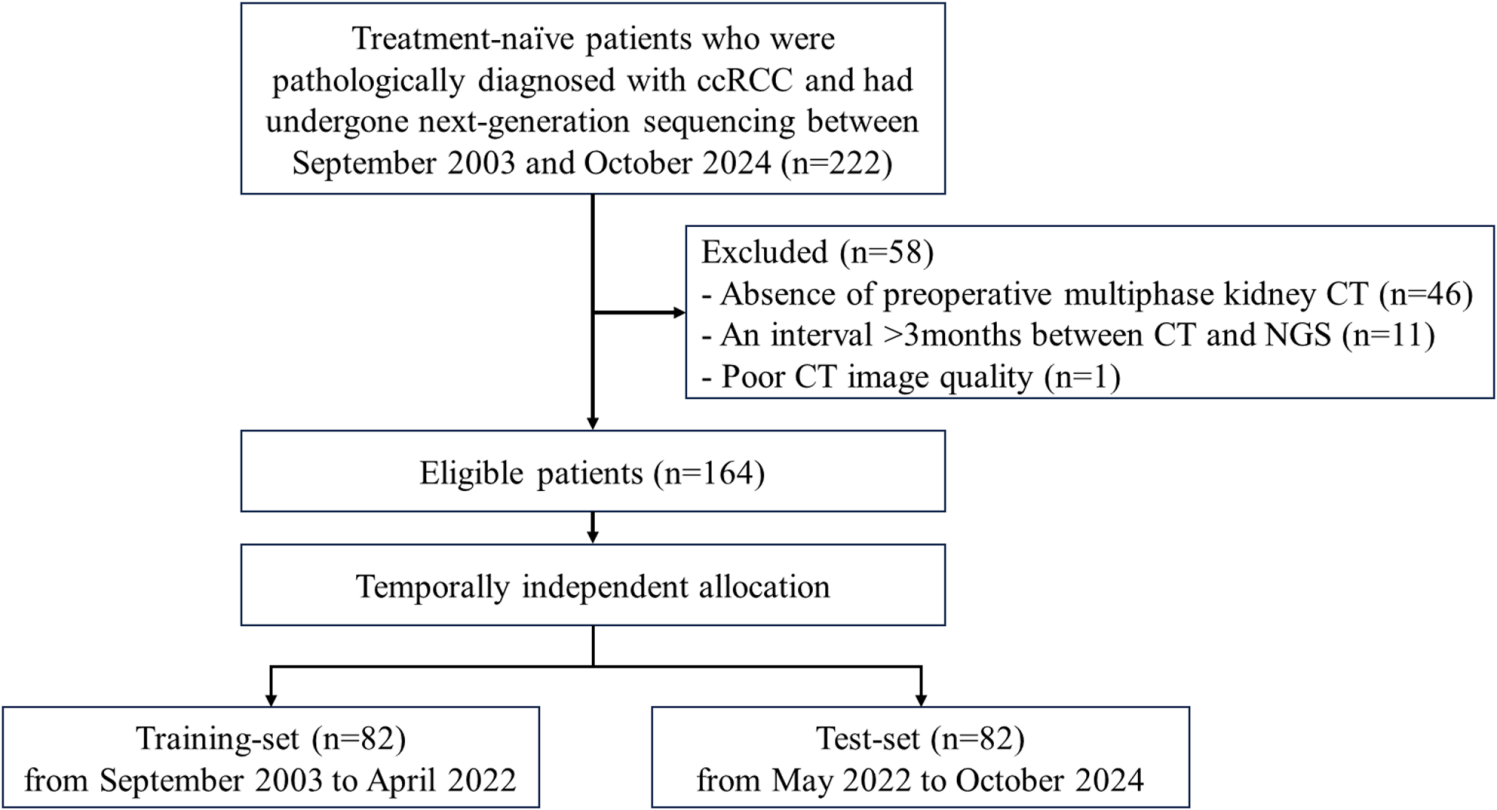
Flow chart summarizing patient selection and allocation to the training and test-sets. ccRCC, clear cell renal cell carcinoma; CT, computed tomography; NGS, next-generation sequencing

This retrospective observational cohort study was conducted in accordance with the Declaration of Helsinki and approved by the Institutional Review Board (IRB) of Severance Hospital (IRB No. 4-2025-0901). Using electronic medical records, we identified 222 treatment-naive patients who were pathologically diagnosed with ccRCC via biopsy or surgical specimen and had undergone NGS between September 2003 and October 2024. Exclusion criteria included (1) absence of preoperative multiphase kidney CT, (2) > 3months interval between CT and NGS, and (3) poor CT image quality. After applying these criteria, 164 patients were included in the analysis. Patients were allocated into training and test-sets based on the date of NGS: those with NGS performed from September 2003 to April 2022 were assigned to the training-set, and those from May 2022 to October 2024 to the test-set (Fig. 1). Targeted DNA and RNA sequencing was performed using the TruSight Tumor 170 or TruSight Oncology 500 panel (Illumina, San Diego, CA) for specimens with ≥40% tumor cellularity. Detailed information on sequencing procedures and the analysis of genetic alterations is provided in the Supplementary Materials. The Supplementary Materials also provide further details on sample size calculation for the test-set.

### CT image acquisition and analysis

Multiphase kidney CT was performed using the following helical CT scanners: Discovery CT 750 HD, LightSpeed VCT, Revolution EVO, or Revolution CT (GE Healthcare); iCT256 (Philips Healthcare); or Somatom Force, Definition Flash, or Definition AS+ (Siemens Healthcare). CT images were acquired during the unenhanced phase, corticomedullary phase (CMP), nephrographic phase (NP), and excretory phase (EP), with breath-holding during each phase. The CT protocol included axial imaging, 100 kVP, variable tube current, and a section thickness of 3 mm. After intravenous administration of 100–150 mL of a nonionic contrast agent (Xenetix 300, Guerbet), dosed according to body weight and delivered using a power injector at a rate of 3–4 mL/s, a bolus-tracking technique was used to determine the start of CMP imaging (range, 20–35 s). NP and EP were obtained at 60–70 s and 2–3 min, respectively, after contrast injection.

Two board-certified radiologists, each with 4 years of experience interpreting genitourinary CT images, independently performed all qualitative image analyses, blinded to the pathologic and genomic data. The definitions of the various image features analyzed are summarized in Supplementary Table S1. Definitions for the attenuation score and tumor heterogeneity score were adapted from those used in a five-tiered renal CT algorithm [21]. Definitions for the other features were based on previous studies [19, 22]. Radiologic tumor, nodal, and metastasis staging was performed according to the eighth edition of the American Joint Committee on Cancer (AJCC) TNM classification [4]. After one month of independent review, the two radiologists met to draw consensus data, which was used for the final analysis.

### Radiologic clustering analysis

To identify intrinsic radiologic subtypes of ccRCC patients, we applied consensus clustering, an unsupervised method for subtype discovery, using the “CancerSubtypes” R package [23]. Specifically, we employed partitioning around medoid with the Euclidean distance metric to classify patients into subtypes. For each iteration, 80% of the patients were randomly sampled, and this process was repeated 1000 times to generate a consensus matrix. We evaluated cluster solutions ranging from two to six subtypes. The optimal number of clusters was determined based on multiple criteria: changes in the area under the cumulative distribution function (CDF) curve between successive values of k, visual inspection of consensus heatmaps for cluster separation, and the average silhouette width of the resulting clusters. The training-set data was used to generate radiologic clusters.

### Study endpoints

The primary endpoint was 5-year cause-specific PFS among ccRCC patients classified into different radiologic clusters. Secondary endpoints included 5-year overall survival (OS) and the associations between imaging features and genomic alterations. Cause-specific PFS was defined as the time from diagnosis to the date of ccRCC progression, death due to ccRCC, or the last follow-up [24]. OS was defined as the time from diagnosis to death from any cause.

### Statistical analysis

Quantitative variables are presented as medians and 25th-75th percentiles, whereas qualitative variables are presented as numbers and percentages. Comparisons of patient characteristics were conducted using the Mann–Whitney U test and chi-square test for categorical variables, unless more than 20% of cells had expected frequencies < 5, in which Fisher’s exact test was used. Inter-reader agreement was assessed using Cohen’s kappa coefficient for categorical variables and intraclass correlation coefficient (ICC) for continuous variables. A kappa statistic of 0.8–1.0, 0.6–0.79, 0.40–0.59, 0.2–0.39, and 0–0.19 was considered excellent, good, moderate, fair, and poor agreement, respectively. ICC values of 0.90-1.00, 0.75–0.89, 0.50–0.74, and < 0.50 were considered excellent, good, moderate, and poor reliability, respectively. Univariable and multivariable logistic regressions analyses were performed on the training-set to identify independent predictors of cluster, C1. A stepwise backward procedure for variable selection was applied to the multivariable logistic regression analysis, and variables with a variance inflation factor (VIF) greater than 5 were excluded to minimize multicollinearity. A nomogram was constructed based on the selected predictive variables using “rms” R package. The Hosmer-Leme-show test and calibration plots were used to test the performance of the nomogram. The optimal cutoff value for the nomogram score in predicting cluster C1 was identified using Youden’s index. The diagnostic performance of individual nomogram components as well as the total score was assessed on the test-set by calculating sensitivity, specificity, positive predictive value (PPV), negative predictive value (NPV), and their corresponding 95% confidence intervals (CIs). The 5-year cause-specific PFS was compared between ccRCC patients classified into different radiologic clusters for training-, test-, and all data. Survival differences were estimated by Kaplan-Meier (K-M) curves using the log-rank test. Univariable and multivariable Cox regression analyses were conducted on the training-set to identify independent predictors of 5-year cause-specific PFS. Multiple prognostic models were developed using various combinations of variables that were significant in the multivariable analysis. The predictive performance of each model was assessed on the test-set using the concordance index (C-index) and time-dependent receiver operating characteristic (ROC) curves. Model calibration was evaluated using calibration plots, and predictive error was quantified by the integrated Brier score (IBS), calculated via the “Boot632plus” resampling method. All analyses were performed using R version 3.4.3 (http://www.r-project.org/). For all analyses, a two-sided P-value of < 0.05 was considered statistically significant.

## Results

### Study patients’ characteristics

A total of 164 patients were included across both training and test-sets, and their clinical, pathologic, radiologic and genomic characteristics are summarized in Table 1 and Supplementary Table S2. Except for Furhman grade, no significant differences were observed between the two sets in these characteristics. The median interval between preoperative CT and NGS was 42 days.

**Table 1:** Clinical, pathologic, and radiologic characteristics of training- and test-sets

Variable	Data available	Training-set (*n* = 82, 50%)	Test-set (*n* = 82, 50%)	*p*-value
Age (years)	164	59.0 (50.0-65.8)	60.5 (51.3–67.0)	0.450
Sex (men)	164	62 (75.6)	62 (75.6)	0.999
BMI (kg/m^2^)	164	23.7 (22.1–26.4)	24.4 (21.5–26.1)	0.970
Operation/biopsy	164			0.117
Renal biopsy		17 (20.7)	28 (34.1)	
Non-renal biopsy		1 (1.2)	0 (0)	
PN		12 (14.6)	14 (17.1)	
RN		52 (63.4)	40 (48.8)	
Pathologic findings				
Furhman grade	140			0.003
1		0 (0)	1 (1.5)	
2		19 (25.3)	16 (24.6)	
3		43 (57.3)	24 (36.9)	
4		13 (17.3)	24 (36.9)	
LNVI	114			0.093
Absent		48 (76.2)	37 (72.5)	
Present		15 (23.8)	14 (27.5)	
Radiologic findings (consensus)				
Tumor size (cm)	164	6.5 (4.4–9.4)	7.0 (4.0-9.8)	0.934
Side	164			0.999
Left		41 (50.0)	41 (50.0)	
Right		41 (50.0)	40 (48.8)	
Both		0 (0)	1 (1.2)	
Attenuation score	164	0.59 (0.44–0.84)	0.47 (0.34–0.63)	< 0.001
Tumor heterogeneity score	164			0.088
1		3 (3.7)	2 (2.4)	
2		4 (4.9)	5 (6.1)	
3		23 (28.0)	23 (28.0)	
4		33 (40.2)	19 (23.2)	
5		19 (23.2)	33 (40.2)	
Multicystic appearance	164	4 (4.9)	3 (3.7)	0.999
Non-smooth margin	164	40 (48.8)	54 (65.9)	0.040
Growth pattern	164			0.009
Endophytic		0 (0)	7 (8.5)	
< 50% exophytic		27 (32.9)	18 (22.0)	
≥50% exophytic		55 (67.1)	57 (69.5)	
Nodular enhancement	164	65 (79.3)	53 (64.6)	0.055
Necrosis (for solid tumor)	164	71 (86.6)	72 (87.8)	0.999
Calcification	164	18 (22.0)	17 (20.7)	0.999
Renal sinus extension	164	46 (56.1)	46 (56.1)	0.999
Renal vein invasion	164	22 (26.8)	18 (22.0)	0.586
Renal vein tumor thrombosis	164	18 (22.0)	17 (20.7)	0.999
Collecting duct invasion	164	15 (18.3)	14 (17.1)	0.999
Intratumoral vessel	164	47 (57.3)	43 (52.4)	0.638
cT stage	164			0.493
1		26 (31.7)	28 (34.1)	
2		12 (14.6)	7 (8.5)	
3		10 (12.2)	15 (18.3)	
4		34 (41.5)	32 (39.0)	
cN1		21 (25.6)	16 (19.5)	0.455
cM1		26 (31.7)	28 (34.1)	0.868
Adjuvant treatment	164			
None		15 (18.3)	8 (9.8)	
TKI		5 (6.1)	42 (51.2)	
IO + IO		34 (41.5)	11 (13.4)	
TKI + IO		28 (34.1)	21 (25.6)	

Data is presented as either median (25th-75th percentile) or number (percentage)

Abbreviations: BMI-body mass index; IO, immune-oncology; LNVI, lymphovascular or neurovascular invasion; PN-partial nephrectomy; T-tumor; TKI, tyrosine kinase inhibitor; RN-radical nephrectomy

### Associations between imaging features and genomic alterations

Inter-reader agreement, summarized in Supplementary Table S3, was fair for tumor heterogeneity score, multicystic appearance, non-smooth margin, nodular enhancement, renal sinus extension, and intratumoral vessel, while all remaining imaging features showed moderate to good reproducibility. Several imaging features showed significant associations with genomic alterations. PBRM1 mutations were linked to higher attenuation scores (*p* = 0.017), and MTOR mutations to multi-cystic appearance (*p* = 0.022) (Supplementary Table S4 and Supplementary Fig S1). Nodular enhancement was more frequent in tumors with 3p (*p* = 0.011) and 7q alterations (*p* = 0.025). Necrosis was associated with mutations or CNVs in VHL (*p* = 0.008), 5q (*p* = 0.015), and 8q gain (*p* = 0.025). Renal vein invasion and thrombosis were more frequent in tumors with 7q alterations (*p* = 0.003 and *p* = 0.026, respectively). Collecting duct invasion was more common in SETD2-mutant tumors (*p* = 0.029). Intratumoral vessels were associated with PTEN, 5p, and 5q alterations (*p* = 0.023, 0.033, and 0.014, respectively). Distant metastasis was less common with 3p alterations (*p* = 0.031), but more frequent with 8q gain (*p* = 0.015). Finally, tumors with 8q gain were significantly smaller (median size, 3.7 vs. 7.0 cm, *p* = 0.007).

### Radiologic subtypes in the training-set

**Fig. 2:**
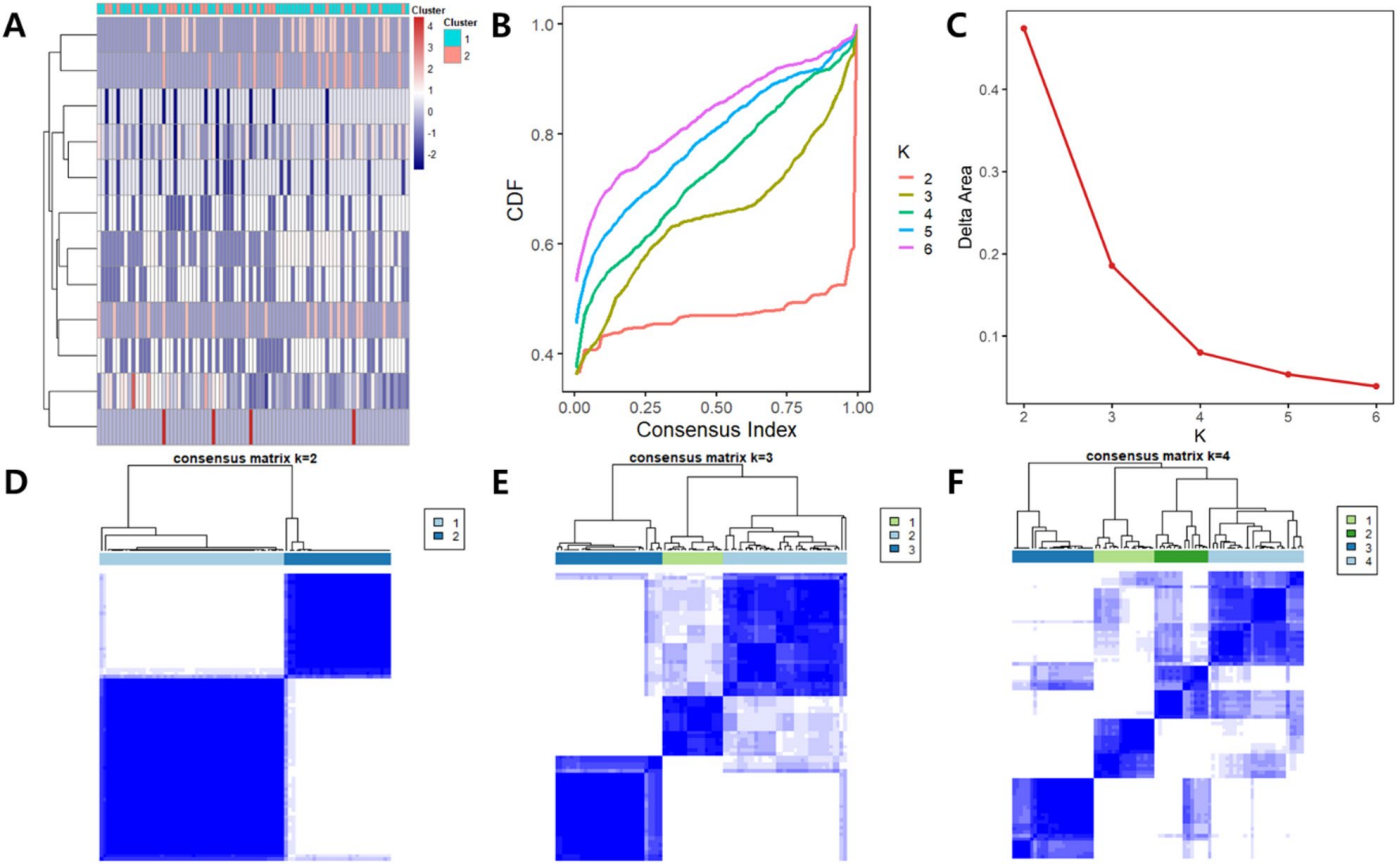
Consensus clustering for radiologic stratification of ccRCC patients. (**A**) Heatmap of CT imaging features used for supervised consensus clustering. The top bar indicates resulting cluster assignments for k = 2. (**B**) Cumulative distribution function (CDF) plots for k = 2 to k = 6, showing increased clustering stability with higher k, but diminished returns beyond k = 2. (**C**) Delta area plot demonstrating the relative change in area under the CDF curve. The largest gain occurs at k = 2, indicating this as the optimal number of clusters. (**D**) Consensus matrix heatmap for k = 2, showing clear separation into two stable clusters. (**E**) Consensus matrix heatmap for k = 3, demonstrating reduced stability and more diffuse boundaries between clusters. (**F**) Consensus matrix heatmap for k-4, with further decreased cluster stability and less distinct separation among groups

A total of 18 imaging features were analyzed for each tumor. Based on consensus clustering of these features, a two-cluster solution was identified as the optimal solution. The consensus CDF and delta plot demonstrated greatest relative change between k = 2 and k = 3 (Fig. 2B and C). The consensus matrix heatmap for k = 2 showed the highest within-cluster consensus and the most distinct separation between clusters (Fig. 2D-F). Furthermore, the average silhouette distance was highest for k = 2 (0.291) compared to k = 3 (0.179), k = 4 (0.188), k = 5 (0.194), and k = 6 (0.191). Thus, 164 patients were divided into two clusters: 106 patients (65%) in cluster C1 and 58 patients (35%) in cluster C2.

### Distinctive characteristics of radiologic subtype

Clusters C1 and C2 exhibited distinct clinicopathologic and radiologic profiles (Table 2). Overall, cluster C1 consistently demonstrated more aggressive features, including larger tumor size, higher heterogeneity scores, non-smooth margins, ≥50% exophytic growth patterns, nodular enhancement, necrosis, renal sinus extension, renal vein invasion and tumor thrombosis, collecting duct invasion, presence of intratumoral vessels, and higher T-stage (all *p* < 0.05). Reflecting this, radical nephrectomy was more frequent in C1 across both training and test-sets. Lymphovascular or neurovascular invasion (LNVI) was also significantly more common in C1 within the training-set (*p* = 0.009), with a similar trend in the test-set (*p* = 0.136). Conversely, distant metastasis was more frequent in C1 in the test-set (*p* = 0.029).

**Table 2:** Clinical, pathologic, and radiologic characteristics of cluster C1 and C2 in training- and test-sets

Variable	Data	Training-set (*n* = 82, 50%)		*p*-value	Data	Test-set (*n* = 82, 50%)		*p*-value
		Cluster 1-C1 (*n* = 52–63%)	Cluster 2-C2 (*n* = 30–37%)			Cluster 1-C1 (*n* = 54–66%)	Cluster 2-C2 (*n* = 28–34%)	
Age (years)	82	59.5 (48.8–67.3)	59.0 (51.3–65.0)	0.679	82	62.5 (51.3–67.8)	58.5 (52.0-65.3)	0.528
Sex (men)	82	39 (75.0)	23 (76.7)	0.999	82	40 (74.1)	22 (78.6)	0.789
BMI (kg/m^2^)	82	23.6 (22.0-26.1)	23.76 (22.5–27.0)	0.494	82	24.3 (21.4–26.3)	24.5 (21.8–25.9)	0.926
Operation/biopsy	82			< 0.001	82			0.040
Renal biopsy		9 (17.3)	8 (26.7)			21 (38.9)	7 (25.0)	
Non-renal biopsy		1 (1.9)	0 (0)			0 (0)	0 (0)	
PN		2 (3.8)	10 (33.3)			5 (9.3)	9 (32.1)	
RN		40 (76.9)	12 (40.0)			28 (51.9)	12 (42.9)	
Pathologic findings								
Furhman grade	75			0.692	65			0.086
1		0 (0)	0 (0)			1 (2.4)	0 (0)	
2		12 (25.5)	7 (25.0)			6 (14.6)	10 (41.7)	
3		25 (53.2)	18 (64.3)			18 (43.9)	6 (25.0)	
4		10 (21.3)	3 (10.7)			16 (39.0)	8 (33.3)	
LNVI	63			0.009	51			0.136
Absent		25 (64.1)	23 (95.8)			20 (64.5)	17 (85.0)	
Present		14 (35.9)	1 (4.2)			11 (35.5)	3 (15.0)	
Radiologic findings (consensus)								
Tumor size (cm)	82	8.8 (6.5–10.6)	4.1 (3.0-5.4)	< 0.001	82	9.0 (6.2–10.3)	3.5 (2.6–4.4)	< 0.001
Side	82			0.999	82			0.033
Left		26 (50.0)	15 (50.0)			23 (42.6)	18 (64.3)	
Right		26 (50.0)	15 (50.0)			31 (57.4)	9 (32.1)	
Both		0 (0)	0 (0)			0 (0)	1 (3.6)	
Attenuation score	82	0.59 (0.44–0.86)	0.60 (0.45–0.82)	0.714	82	0.43 (0.33–0.57)	0.55 (0.40–0.69)	0.083
Tumor heterogeneity score	82			< 0.001	82			< 0.001
1		0 (0)	3 (10.0)			0 (0)	2 (7.1)	
2		0 (0)	4 (13.3)			1 (1.9)	4 (14.3)	
3		6 (11.5)	17 (56.7)			7 (13.0)	16 (57.1)	
4		27 (51.9)	6 (20.0)			14 (25.9)	5 (17.9)	
5		19 (36.5)	0 (0)			32 (59.3)	1 (3.6)	
Multicystic appearance	82	2 (3.8)	2 (6.7)	0.621	82	1 (1.9)	2 (7.1)	0.267
Non-smooth margin	82	39 (75.0)	1 (3.3)	< 0.001	82	47 (87.0)	7 (25.0)	< 0.001
Growth pattern	82			< 0.001	82			< 0.001
Endophytic	82	0 (0)	0 (0)		82	0 (0)	7 (25.0)	
< 50% exophytic	82	5 (9.6)	22 (73.3)		82	3 (5.6)	15 (53.6)	
≥50% exophytic	82	47 (90.4)	8 (26.7)		82	51 (94.4)	6 (21.4)	
Nodular enhancement	82	50 (96.2)	15 (50.0)	< 0.001	82	43 (79.6)	10 (35.7)	< 0.001
Necrosis (for solid tumor)	82	51 (98.1)	20 (66.7)	< 0.001	82	53 (98.1)	19 (67.9)	< 0.001
Calcification	82	17 (32.7)	1 (3.3)	0.002	82	14 (25.9)	3 (10.7)	0.153
Renal sinus extension	82	42 (80.8)	4 (13.3)	< 0.001	82	41 (75.9)	5 (17.9)	< 0.001
Renal vein invasion	82	21 (40.4)	1 (3.3)	< 0.001	82	18 (33.3)	0 (0)	< 0.001
Renal vein tumor thrombosis	82	17 (32.7)	1 (3.3)	0.002	82	17 (31.5)	0 (0)	< 0.001
Collecting duct invasion	82	14 (26.9)	1 (3.3)	0.007	82	13 (24.1)	1 (3.6)	0.028
Intratumoral vessel	82	40 (76.9)	7 (23.3)	< 0.001	82	37 (68.5)	6 (21.4)	< 0.001
cT stage	82			< 0.001	82			< 0.001
1		7 (13.5)	19 (63.3)			9 (16.7)	19 (67.9)	
2		11 (21.2)	1 (3.3)			4 (7.4)	3 (10.7)	
3		10 (19.2)	0 (0)			15 (27.8)	0 (0)	
4		24 (46.2)	10 (33.3)			26 (48.1)	6 (21.4)	
cN1	82	16 (30.8)	5 (16.7)	0.196	82	13 (24.1)	3 (10.7)	0.239
cM1	82	18 (34.6)	8 (26.7)	0.623	82	23 (42.6)	5 (17.9)	0.029
Adjuvant treatment	82			0.085	82			0.078
None		5 (9.6)	3 (10.0)			6 (11.1)	9 (32.1)	
TKI		23 (44.2)	19 (63.3)			3 (5.6)	2 (7.1)	
IO + IO		6 (11.5)	5 (16.7)			23 (42.6)	11 (39.3)	
TKI + IO		18 (34.6)	3 (10.0)			22 (40.7)	6 (21.4)	

Data is presented as either median (25th-75th percentile) or number (percentage)

Abbreviations: BMI-body mass index; IO, immune-oncology; LNVI, lymphovascular or neurovascular invasion; PN-partial nephrectomy; T-tumor; RN-radical nephrectomy; TKI, tyrosine kinase inhibitor

**Fig. 3:**
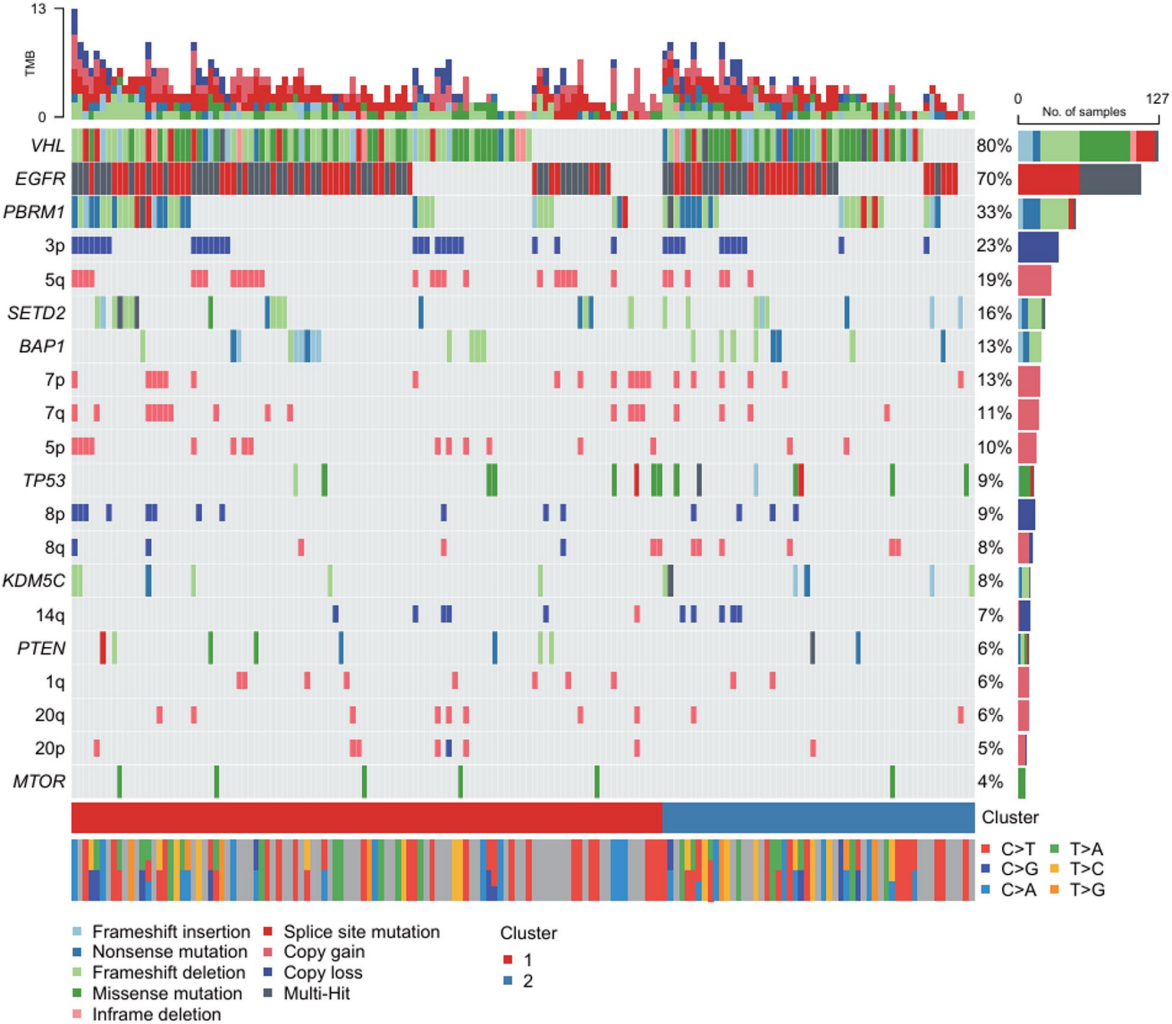
Oncoplot of genetic alterations across ccRCC patients, stratified by radiologic cluster. The top bar plot shows tumor mutational burden (TMB) for each patient. The bottom bar indicates radiologic cluster assignment (Cluster 1 in red, Cluster 2 in blue)

**Fig. 4:**
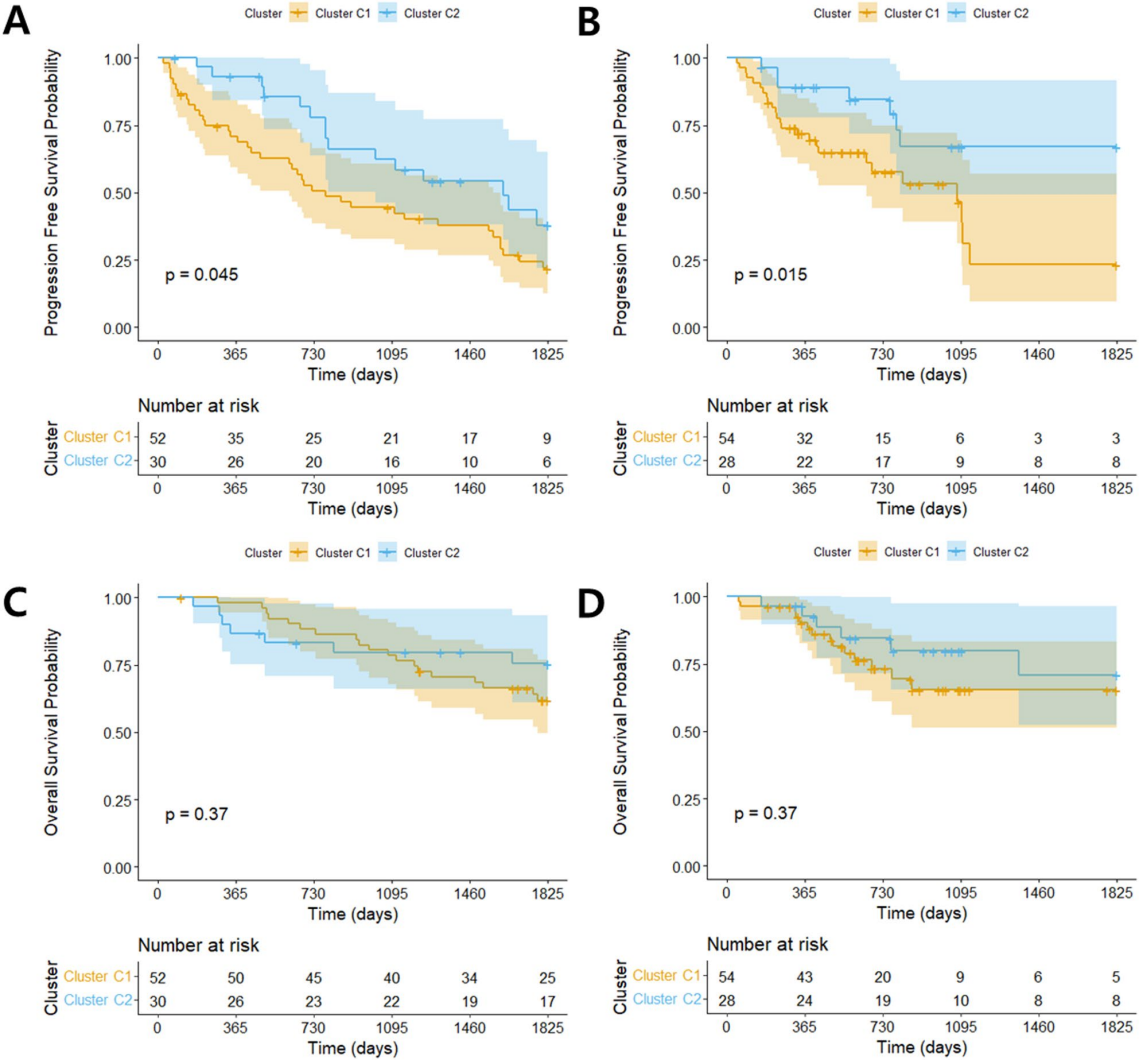
Kaplan-Meier survival analysis stratified by radiologic clusters (C1 vs. C2) in ccRCC patients. Shaded areas represent 95% confidence interval. (**A**-**B**) Cause-specific progression-free survival (PFS) curves for cluster C1 (orange) and C2 (blue) in the training (**A**), and test (**B**). In both sets, cluster C1 was associated with significantly poorer 5-year cause-specific PFS. (**C**-**D**) Overall survival (OS) curves for the training (**D**), and test (**E**). No statistically significant difference in OS was observed between clusters, although a trend toward worse survival in cluster C1 was noted

Despite these phenotypic differences, genomic alterations were largely similar between clusters (Supplementary Table S5). Only 7q gain was significantly more frequent in C1 in the training-set (*p* = 0.023); this was not replicated in the test-set. The oncogenomic heatmap for all patients are shown in Fig. 3. However, K-M analysis revealed significantly shorter 5-year cause-specific PFS for cluster C1 across all datasets (Fig. 4A, training-set, median cause-specific PFS of C1 [95% CI]:951.0 [763.2-1133.8] days vs. C2: 1298.8 [1071.6-1506.7] days, *p* = 0.045; Fig. 4B, test-set, C1: 919.1 [701.9-1142.5] days vs. C2: 1411.8 [1140.1-1652.5] days, *p* = 0.015), while no significant difference in 5-year OS was observed (Fig. 4C-D). Cox proportional hazards analysis further confirmed significantly higher cause-specific progression risk in cluster C1 (training-set: hazard ratio [HR]: 1.83 [95% CI, 1.01–3.33], *p* = 0.048; test-set: HR: 2.72 [1.17–6.42], *p* = 0.020), consistent with the K-M results.

### Nomogram for predicting cluster C1

**Fig. 5:**
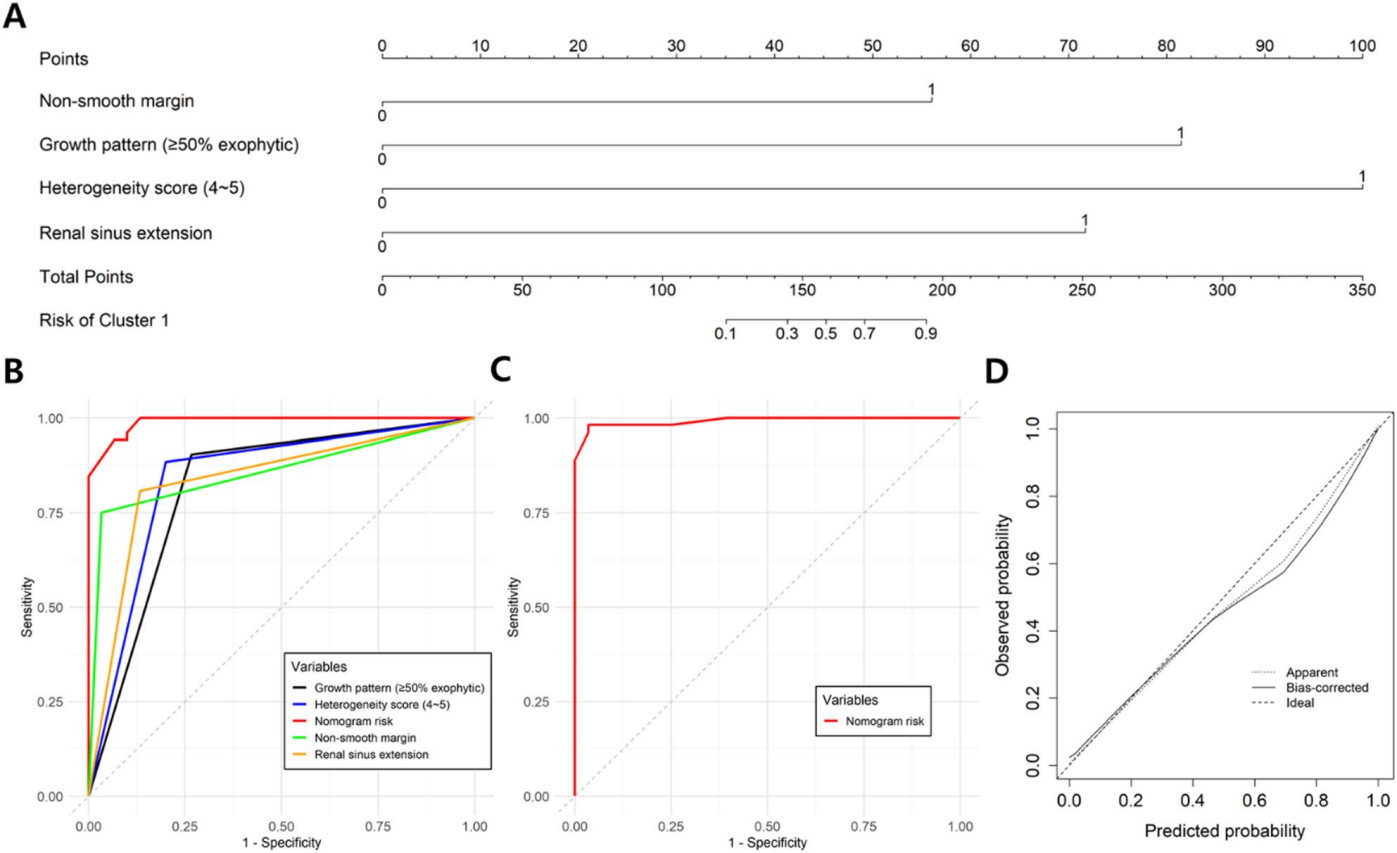
Development and validation of a nomogram-based C1 score for predicting cluster C1 in ccRCC patients. (**A**) Nomogram for calculating the probability of assignment to cluster C1. Total points correspond to the estimated risk of cluster C1. (**B**) Receiver operating characteristic curves showing the diagnostic performance of individual imaging features and nomogram risk score in the training-set. The nomogram demonstrated superior discrimination compared to each individual feature. (**C**) Receiver operating characteristic curve for the nomogram in the test-set. (**D**) Calibration plot of the nomogram in the test-set, showing good agreement between predicted and observed probabilities for cluster C1. Apparent and bias-corrected curves are shown against the ideal reference line

Given the distinct 5-year cause-specific PFS between clusters, a nomogram, hereafter referred to as the C1 score, was developed using age, sex, and imaging features to identify cases belonging to cluster C1. Multivariate logistic regression identified heterogeneity score ≥4 (*p* < 0.001), non-smooth margin (*p* = 0.006), ≥50% exophytic growth pattern (*p* < 0.001), and renal sinus extension (*p* = 0.026) as independent predictors of cluster C1 (Supplementary Table S6). The C1 score (Fig. 5A) achieved an AUC of 0.990 (95% CI, 0.978-1.000) in the training-set (Fig. 5B), significantly outperforming each individual feature: heterogeneity score ≥4 (AUC of 0.842, 95% CI, 0.748–0.925), non-smooth margin (AUC of 0.858, 95% CI, 0.788–0.927), ≥50% exophytic growth pattern (AUC of 0.819, 95% CI, 0.721–0.901), and renal sinus extension (AUC of 0.837, 95% CI, 0.745–0.914) (all DeLong test, *p* < 0.001). In the test-set, the C1 score retained high performance with an AUC of 0.992 (95% CI, 0.979-1.000) (Fig. 5C). The optimal cutoff of the C1 score was 0.75, as determined by the Youden index, yielding a sensitivity of 94.2% and specificity of 93.3% in the training-set, and 96.3% and 96.4%, respectively in the test-set (Table 3). The Hosme-Leme-show test validated the good-fit of C2 score for the test-set (X^2^ = 5.481, *p* = 0.710) as shown in Fig. 5D.

**Table 3:** Diagnostic performance of the nomogram C1 score and its individual components for predicting cluster C1

	Sensitivity (95%CI)	Specificity (95% CI)	Positive predictive value (95% CI)	Negative predictive value (95% CI)
**Training-set**				
Components of Cluster C1 score				
Heterogeneity score, 4 ~ 5	88.5% (79.8–97.1%)	80.0% (65.7–94.3%)	88.5% (79.8–97.1%)	80.0% (65.7–94.3%)
Non-smooth margin	75.0% (63.2–86.8%)	96.7% (90.2–100%)	97.5% (92.7–100%)	69.0% (55.1–83.0%)
Growth pattern, ≥50% exophytic	90.4% (82.4–98.4%)	73.3% (57.5–89.2%)	85.5% (76.1–94.8%)	81.5% (66.8–96.1%)
Renal sinus extension	80.8% (70.1–91.5%)	86.7% (74.5–98.8%)	91.3% (83.2–99.4%)	72.2% (57.6–86.9%)
C1 score ≥0.75	94.2% (87.9–100%)	93.3% (84.4–100%)	96.1% (90.8–100%)	90.3% (79.9–100%)
**Test-set**				
Components of Cluster C1 score				
Heterogeneity score, 4 ~ 5	85.2% (75.7–94.7%)	78.6% (63.4–93.8%)	88.5% (79.8–97.1%)	73.3% (57.5–89.2%)
Non-smooth margin	87.0% (78.1–96.0%)	75.0% (59.0–91.0%)	87.0% (78.1–96.0%)	75.0% (59.0–91.0%)
Growth pattern, ≥50% exophytic	94.4% (88.3–100%)	78.6% (63.4–93.8%)	89.5% (81.5–97.4%)	88.0% (75.3–100%)
Renal sinus extension	75.9% (64.5–87.3%)	82.1% (68.0-96.3%)	89.1% (80.1–98.1%)	63.9% (48.2–79.6%)
C1 score ≥0.75	96.3% (91.3–100%)	96.4% (89.6–100%)	98.1% (94.5–100%)	93.1% (83.9–100%)
**All-set**				
Components of Cluster C1 score				
Heterogeneity score, 4 ~ 5	86.8% (80.3–93.2%)	79.3% (68.9–89.7%)	88.5% (82.3–94.6%	76.7% (66.0-87.4%)
Non-smooth margin	81.1% (73.7–88.6%)	86.2% (77.3–95.1%)	91.5% (85.8–97.1%)	71.4% (60.8–82.0%)
Growth pattern, ≥50% exophytic	92.5% (87.4–97.5%)	75.9% (64.8–86.9%)	87.5% (81.4–93.6%)	84.6% (74.8–94.4%)
Renal sinus extension	78.3% (70.5–86.1%)	84.5% (75.2–93.8%)	90.2% (84.1–96.3%)	68.1% (57.3–78.8%)
C1 score ≥0.75	95.3% (91.2–99.3%)	94.8% (89.1–100%)	97.1% (93.9–100%)	91.7% (84.7–98.7%)

Abbreviations: CI, confidence interval

### Model predictions of 5-year cause-specific PFS

Multivariate cox regression analysis identified C1 score ≥0.75 (HR, 1.85; 95% CI, 1.11–3.07, *p* = 0.018), nodal metastasis (HR, 2.26; 95% CI, 1.32–3.86; *p* = 0.003), and distant metastasis (HR, 2.22; 95% CI, 1.25–3.96; *p* = 0.007), and BAP1 mutation (HR, 1.98; 95% CI, 1.10–3.58; *p* = 0.023) as independent predictors of 5-year cause-specific PFS (Table 4). Based on these findings, four models were constructed to predict 5-year cause-specific PFS: (1) Model 1: C1 score≥0.75 only; (2) Model 2: C1 score, nodal metastasis, and distant metastasis (imaging-only model); (3) Model 3: BAP1 mutation only (genomic-only model); and (4) Model 4: all independent predictors (combined model).

**Table 4:** Univariate and multivariate Cox regression analyses for risk of progression within 5-years

Variables	Univariate analysis		Multivariate analysis	
	Hazard ratio (95% CI)	p-value	Hazard ratio (95% CI)	p-value
Age ≥65years	0.80 (0.34–1.86)	0.596		
Sex (men)	0.75 (0.47–1.19)	0.221		
BMI (kg/m^2^)	0.99 (0.93–1.06)	0.843		
Tumor size (cm)	1.03 (0.98–1.08)	0.222		
C1 score ≥0.75	1.97 (1.24–3.15)	0.004	1.85 (1.11–3.07)	0.018
cT stage				
cT1-2	(ref.)		(ref.)	
cT3-4	3.82 (2.40–6.10)	< 0.001	1.56 (0.81–3.00)	0.180
cN1	3.66 (2.28–5.87)	< 0.001	2.26 (1.32–3.86)	0.003
cM1	4.12 (2.61–6.50)	< 0.001	2.22 (1.25–3.96)	0.007
Operation/biopsy				
Biopsy	(ref.)		(ref.)	
PN	0.14 (0.05–0.36)	< 0.001	0.63 (0.11–3.67)	0.606
RN	0.24 (0.10–0.53)	< 0.001	0.27 (0.11–0.66)	0.005
Adjuvant treatment				
None	(ref.)		(ref.)	
TKI	9.40 (2.26–39.14)	0.002	6.53 (1.55–27.55)	0.011
IO + IO	9.40 (2.21–39.94)	0.002	5.91 (1.36–25.63)	0.018
IO + TKI	8.90 (2.11–37.54)	0.003	6.35 (1.49–27.03)	0.012
Nucleotide substitution mutations				
VHL	0.89 (0.53–1.48)	0.641		
EGFR splice	1.01 (0.64–1.60)	0.965		
PBRM1	0.71 (0.45–1.15)	0.164		
SETD2	1.26 (0.72–2.20)	0.423		
BAP1	1.94 (1.11–3.41)	0.020	1.98 (1.10–3.58)	0.023
TP53	1.50 (0.78–2.91)	0.228		
KDM5C	1.23 (0.53–2.82)	0.628		
TERT	1.41 (0.65–3.06)	0.386		
MTOR	0.86 (0.21–3.50)	0.830		
PTEN	1.38 (0.64–3.00)	0.411		
Copy-number variations				
8q gain	1.50 (0.61–3.71)	0.381		
8q loss	2.81 (0.68–11.60)	0.152		
14q gain	0.00 (0.00–Inf)	0.995		
14q loss	0.58 (0.21–1.60)	0.297		
20p gain	0.65 (0.16–2.65)	0.549		
20p loss	5.67 (0.77–41.82)	0.089		
TMB (mut/MB)	1.00 (0.93–1.08)	0.942		
MSI (%)	0.99 (0.87–1.14)	0.934		

Abbreviations: BAP1, BRCA21 associated protein; BMI, body mass index; CI, confidence interval; EGFR splice, epidermal growth factor receptor slice variant; IO, immune-oncology; KDM5C, lysine demethylase 5 C; M, distant metastasis; MTOR, mechanistic target of rapamycin; MSI, microsatellite instability; N, nodal; PBRM1, polybromo 1; PN, partial nephrectomy; PTEN, phosphatase and tensin homolog; RN, radical nephrectomy; SETD2, SET domain containing 2; T, tumor; TERT, telomerase reverse transcriptase; TKI, tyrosine kinase inhibitor; TMB, tumor mutational burden; TP53, tumor protein p53; VHL, Von Hippel-Lindau tumor suppressor

The performances of these models in the test-set are summarized in Table 5. Model 1 demonstrated modest performance (AUC, 0.647), outperforming Model 3 based solely on BAP1 mutation (AUC 0.526). Model 2, which incorporated nodal and distant metastases alongside the C1 score, showed improved performance (AUC 0.702). Model 4, combining all predictors, achieved the highest predictive accuracy (AUC 0.710). Time-dependent ROC curves and prediction error curves for these models in the test-set are presented in Supplementary Fig. S2.

**Table 5:** Prognostic performance of various models for predicting 5-year cause-specific PFS

Models	C-Index (95% CI)	5-year AUC	IBS	*p*-value
Model 1 (C1 score ≥0.75)	0.653 (0.605–0.701)	0.647	0.141	< 0.001
Model 2 (C1 score ≥0.75, cN1, cM1)	0.667 (0.587–0.749)	0.702	0.122	< 0.001
Model 3 (BAP1 mutation)	0.456 (0.389–0.498)	0.526	0.139	(ref.)
Model 4 (C1 score ≥0.75, cN1, cM1, BAP1 mutation)	0.670 (0.580–0.732)	0.710	0.114	< 0.001

Abbreviations: AUC, time-dependent area under the receiver operating characteristic curve; BAP1, BRCA21 associated protein; C-index, concordance index; CI, confidence interval; IBS, integrated Brier score; N, nodal; M, distant metastasis

## Discussion

In this study, we applied unsupervised clustering to classify ccRCCs based on CT imaging features, identifying two distinct subtypes: clusters C1 and C2. Cluster C1, characterized by more aggressive clinico-radiologic characteristics, was independently associated with poorer 5-year cause-specific PFS. For clinical applicability, we developed a nomogram based C1 score using key imaging features associated with cluster C1. The C1 score alone demonstrated modest predictive performance for 5-year cause-specific PFS, which was further improved by incorporating nodal and distant metastasis status and BAP1 mutation status.

Radiological morphology enables a comprehensive assessment of the entire tumor, addressing the limitation of partial sampling inherent in pathology. Prior studies have linked features such as larger tumor size [25–27], renal vein invasion [28], lymph node metastasis [27], and distant metastasis [26] with poor survival, though many are already incorporated into staging systems [4, 29]. Beyond staging-related features, tumor necrosis [27, 30, 31], ≥50% tumor contour irregularity [32], and renal sinus extension over perinephritic fat invasion [33, 34] have been proposed as independent predictors of 5-year cause-specific PFS.

Our findings align with these reports. Necrosis and heterogeneity were both associated with cluster C1 in univariable analysis, but only heterogeneity score remained in the final model due to collinearity. Notably, heterogeneity often increases in the presence of necrosis. Moreover, the definition of contour irregularity in earlier study [32] corresponds to our definitions of exophytic growth and non-smooth margin. While renal sinus extension was once thought not to predict disease specific survival [33, 34], more recent evidence show reduced renal sinus fat area correlates with shorter cause-specific PFS [35]. The recurrence of these features through unsupervised clustering supports the role of radiologic characterization in prognostic stratification. Given that necrosis is a key component of models such as SSIGN score [6], and the 2003 Leibovich model [7], its contribution via the heterogeneity score further underscores the value of our classifier. Furthermore, unlike the pathology-dependent SSIGN and 2023 Leibovich scores, which rely on postoperative histologic data, the C1 score provides preoperative, imaging-based risk stratification. As such, it complements these established models by enabling risk assessment before surgery and may help guide treatment planning and surveillance intensity in the preoperative setting.

However, no cluster showed a distinct genetic alteration. Although several significant associations were observed between imaging features and genetic alterations, these involved features not specific to cluster C1. Contrary to a prior finding linking BAP1 mutation, with calcification and ill-defined margins [19], these associations were not observed in our cohort. The original study also noted the need for validation, as many ill-defined tumors lacked BAP1 mutations [19]. In our cohort, BAP1 mutation was the only alteration independently associated with 5-year cause-specific PFS, consistent with previous work [36]; however, no specific feature predicted its presence. Similarly, previously reported associations between BAP1/KDM5C mutations and renal vein invasion [20] were not replicated. In that study, renal vein invasion still occurred in the majority of cases without either mutation, limiting discriminatory utility [20].

Chromosomal instabilities, including losses of 4p, 9p, and 14q, and gains of 7q, 8q, and 20, have been linked to advanced stage and poorer prognosis [37, 38], while gains of 1q, 12q, and 20q, and 9p loss associate with metastasis [39]. In our cohort, 3p loss, 5q gain and 14q loss were most frequent, consistent with an earlier study [38]. However, the overall CNV burden was insufficient to demonstrate significant associations with imaging features or survival, highlighting the need for further investigation.

The strength of our study lies in demonstrating the prognostic value of radiologic clustering. BAP1 mutations (10–15% [40]), and CNVs (21% [41]) occur at modest rates, and offer limited stratification potential compared to radiologic features, which are noninvasive and widely characterizable. While BAP1 mutation may predict immunotherapy response [42], CNVs have yet to be integrated into treatment planning. Recent trials support the use of immune checkpoint inhibitors such as pembrolizumab after nephrectomy and in advanced disease [43, 44]. Notably, tumors with necrosis may be resistant to anti PD-1 therapy due to impaired T-cell function [45]. Our algorithm may thus serve both prognostic and therapeutic purposes.

This study has several limitations. Its single-center retrospective design introduces potential selection bias and limits generalizability. While there are some differences between training and test-sets, these differences may reflect real-world variability. The cohort size was relatively small, as the analysis was restricted to patients with NGS data. The low frequency of certain genetic alterations raises the risk of a type II error, in which true associations may have been missed. In addition, although operation and adjuvant treatment types influence 5-year cause-specific PFS, they were not incorporated into the model because we aimed to develop pre-treatment models based solely on CT features and NGS data obtained prior to operation and/or adjuvant therapy. Furthermore, the absence of immune biomarker data such as PD-1/PD-L1 expression precluded evaluation of their potential interaction with immunotherapy response, which should be addressed in future studies. Finally, heterogenous acquisition parameters across multiple CT scanners may have influence model performance; however, this heterogeneity also supports the model’s robustness across diverse imaging conditions.

## Conclusion

In summary, we developed and validated a nomogram-based C1 score derived from unsupervised clustering of CT features that effectively predict 5-year cause-specific PFS of ccRCC. The C1 score may function as a noninvasive imaging biomarker to identify high-risk patients preoperatively, supporting treatment planning and individualized follow-up.

## Supplementary Information

Below is the link to the electronic supplementary material.

Supplementary Material 1

## Data Availability

The datasets used and/or analysed during the current study are available from the corresponding author on reasonable request.
