# Supplementary material for: Computed tomography-based unsupervised clustering identifies clusters associated with progression free survival in clear cell renal cell carcinoma
Source: Cancer Imaging. 2025 Nov 24;25:140. doi: 10.1186/s40644-025-00958-x (PMC12751771; doi:10.1186/s40644-025-00958-x)
Supplement: Supplementary file 1 — Supplementary Material 1 [file 40644_2025_958_MOESM1_ESM.docx]

**Supplementary Materials**

*Next-generation sequencing*

Tumor samples were obtained through either surgical resection or biopsy. Targeted DNA and RNA sequencing (RNA-seq) was performed using the TruSight Tumor 170 or TruSight Oncology 500 panel (Illumina, San Diego, CA, USA) if the tumor content was estimated to be ≥40% on pathologic review. According to the manufacturer’s protocol, the recommended minimum tumor content for both panels is ≥20%, with a validated lower limit of approximately 10% [1]. A joint consensus recommendation further suggests that the minimum tumor cell content should be at least twice the limit of detection of the assay used; therefore, NGS is performed on specimens with tumor cellularity ≥40% at our institution [2]. A total of 40 ng of DNA and RNA was extracted from formalin-fixed, paraffin-embedded (FFPE) tissue using the AllPrep DNA/RNA FFPE Kit (Qiagen). Library preparation was carried out according to the manufacturer’s protocol. Following hybridization-based target enrichment, paired-end sequencing (2×150 bp) was performed on NextSeq sequencer (Illumina), also according to the manufacturer’s instructions.

*Genetic alterations analysis*

The raw DNA sequencing data were aligned to the human genome reference genome (Genome Research Consortium human build 38, GRCh38) using the Genome Analysis Toolkit [3]. Unique molecular identifier (UMI) tags were annotated using an in-house script, and duplicate reads were marked using *UmiAwareMarkDUplicatesWithMateCigar* (Picard). Variants called by Mutect2 were filtered based on the following in-house criteria: (1) total read depth<100; (2) variant allele frequency <10%; (3) Combined Annotation Dependent Depletion (CADD) phred score <25; and (4) minor allele frequency >0.1% in either the global or East Asian population of the gnomAD database [4]. Pathogenicity of filtered variants called by HaplotypeCaller was assessed according to the guidelines of the American College of Medical Genetics and Genomics and the Association for Molecular Pathology [5].

CNVs were analyzed using CNVkit [6]. Gene fusion candidates were identified using STAR-Fusion [7], and a fusion expression filter was applied, excluding fusions with <0.5 fusion fragments per million total RNA-seq fragments. An oncoplot summarizing the genomic alterations was generated using MAFtools [8].

*Sample size calculation*

The test-set sample size required to independently evaluate the robustness and efficiency of our model was estimated using a sample size calculation method described in a previous study [9]. To evaluate the difference in mean nomogram score between two groups (designated A [Cluster C1] and B [Cluster C2]), we formulated the following hypotheses:

$$H_{0} : \mu_{A}-\mu_{B} =0$$

$$H_{1} : \mu_{A}-\mu_{B} \neq0$$

The sample size and statistical power were determined based on standard formulas for comparing means between two independent groups, where σ^2^ represents the pooled variance, α is the type I error rate, β is the type II error rate, 1-β is the statistical power, and Φ denotes the cumulative distribution function of the standard normal distribution. Here, n and N refer to the sample sizes of the training and test-sets, respectively.

In this study, the training-set included 52 subjects in Cluster C1, and 30 subjects in Cluster C2, with mean nomogram scores of 0.099. and 0.943, respectively. The pooled variance was calculated as 0.334. Based on these values, the minimum required sample sizes in the test-set to achieve 95% power at a two-sided significance level of 0.01 were estimated to be 22.8 cases in Cluster C1 and 13.2 cases in Cluster C2. Our test-set included 54 subjects in Cluster C1 and 28 cases in Cluster C2, which were greater than the minimum required sample sizes.

Supplementary Table S1. Definitions of qualitative imaging features

| Feature No. | Feature Name | Feature Description | Feature Values |
| --- | --- | --- | --- |
| 1 | Tumor size | Maximal cross-sectional tumor diameter (cm) | Qualitative |
| 2 | Side | Tumor side: right, or left | Binary |
| 3 | Attenuation score | [Mean of three ROIs (≥1cm) on the tumor on CMP] / [One ROI (≥1cm) on the ipsilateral renal cortex on CMP] | Qualitative |
| 4 | Tumor heterogeneity score | 5-point Likert scale:  1, completely homogeneous; 2, mostly homogeneous; 3, mixed areas of heterogeneous and heterogeneous components (also described as mixed heterogeneity); 4, mostly heterogenous; and 5, completely heterogeneous. Heterogeneity was assessed on CMP | Categorical |
| 5 | Multi-cystic appearance | Tumor architecture described as either multi-cystic or solid. Cystic tumor defined as tumor with ≥50% cystic component with fluid attenuation values (≤20 Hounsfield Units) and containing one or more well-demarcated cystic spaces, each of which showing at least 25% of their walls as smooth. | Binary |
| 6 | Non-smooth margin | Presence or absence of non-smooth margin. Smooth margin: the mass shows a well-defined border completely covered by a pseudocapsule; Non-smooth margin: the mass shows a lobulated and disrupted pseudocapsule at the interface.  Margin was considered well-defined with >90% of the entire tumor circumference (including the interface with renal parenchyma, sinus and perinephric fat and collecting system) was 'pencil-thin’ sharp when viewed on either NP or EP. If there was a discordance between the two, the phase that showed the most well-defined tumor was used. | Binary |
| 7 | Growth pattern | Growth pattern defined as one of endophytic, <50% exophytic, and ≥50% exophytic. | Categorical |
| 8 | Nodular enhancement | Presence or absence of intratumoral nodular enhancement in NP. | Binary |
| 9 | Necrosis | Presence or absence of areas within the tumor that does not demonstrate contrast enhancement during NP and EP. | Binary |
| 10 | Calcification | Intratumoral calcification (present or absent) | Binary |
| 11 | Renal sinus extension | Presence of absence of renal sinus extension, defined as a non-smooth margin at the tumor-renal sinus interface or a finger-like projection into the renal sinus fat. | Binary |
| 12 | Renal vein invasion | Presence or absence of renal vein invasion, defined as tumor involvement of renal vein on NP. | Binary |
| 13 | Renal vein tumor thrombosis | Presence or absence of renal vein tumor thrombosis, defined as a filling defect in renal vein contiguous with tumor on NP. | Binary |
| 14 | Collecting duct invasion | Presence or absence of collecting system invasion, defined as evidence of a filling defect on EP. | Binary |
| 15 | Intratumoral vessel | Intratumoral arteries: The presence or absence of discrete enhancing arteries within the tumor | Binary |
| 16 | T stage | Tumor stage based on the eighth edition of the American Joint Committee on Cancer (AJCC) TNM classification. | Categorical |
| 17 | N stage | Nodal metastasis: presence of absence of visible regional nodal metastasis, defined as enlarged regional lymph node >1cm in long-axis, or confirmed FDG-uptake on PET-CT. | Binary |
| 18 | M stage | Distant metastasis: presence or absence of visible lesions suspicious for metastasis within the visualized chest or abdomen | Binary |
| Abbreviations: EP, excretory phase; FDG, fludeoxyglucose; CMP, corticomedullary phase; NP, nephrographic phase; PET: positron emission tomography; ROI, region of interest; | | | |

Supplementary Table S2. Genomic profile of training-, and test-sets.

| Variable | Data available | Training-set  (n=82, 50%) | Test-set  (n=82, 50%) | p-value |
| --- | --- | --- | --- | --- |
| Nucleotide substitution mutations | 164 |  |  | 0.999 |
| VHL |  | 62 (75.6) | 65 (79.3) | 0.970 |
| EGFR splice |  | 55 (67.1) | 56 (68.3) | 0.117 |
| PBRM1 |  | 29 (35.4) | 24 (29.3) |  |
| SETD2 |  | 14 (17.1) | 12 (14.6) |  |
| BAP1 |  | 9 (11.0) | 12 (14.6) |  |
| TP53 |  | 7 (8.5) | 8 (9.8) |  |
| KDM5C |  | 6 (7.3) | 6 (7.3) |  |
| TERT |  | 5 (6.1) | 7 (8.5) | 0.003 |
| MTOR |  | 2 (2.4) | 4 (4.9) |  |
| PTEN |  | 4 (4.9) | 6 (7.3) |  |
| Copy-number variations | 164 |  |  |  |
| 1q gain |  | 5 (6.1) | 5 (6.1) |  |
| 3p loss |  | 19 (23.2) | 17 (20.7) | 0.093 |
| 5p gain |  | 7 (8.5) | 9 (11.0) |  |
| 5q gain |  | 19 (23.2) | 11 (13.4) |  |
| 7p gain |  | 10 (12.2) | 10 (12.2) |  |
| 7q gain |  | 9 (11.0) | 9 (11.0) | 0.934 |
| 8p loss |  | 9 (11.0) | 6 (7.3) | 0.999 |
| 8q gain |  | 2 (2.4) | 8 (9.8) |  |
| 8q loss |  | 1 (1.2) | 2 (2.4) |  |
| 14q gain |  | 1 (1.2) | 0 (0) |  |
| 14q loss |  | 4 (4.9) | 6 (7.3) | <0.001 |
| 20p gain |  | 3 (3.7) | 4 (4.9) | 0.088 |
| 20p loss |  | 0 (0) | 1 (1.2) |  |
| 20q gain |  | 5 (6.1) | 5 (6.1) |  |
| TMB (mut/MB) | 164 | 5.5 (3.9, 7.1) | 5.5 (3.9, 7.1) |  |
| MSI (%) | 164 | 2.6 (1.7, 3.4) | 2.4 (1.6, 3.3) |  |
| Data is presented as either median (25th-75th percentile) or number (percentage)  Abbreviations: BAP1, BRCA21 associated protein; EGFR splice, epidermal growth factor receptor slice variant; KDM5C, lysine demethylase 5C; MTOR, mechanistic target of rapamycin; MSI, microsatellite instability; PBRM1, polybromo 1; PTEN, phosphatase and tensin homolog; SETD2, SET domain containing 2; TERT, telomerase reverse transcriptase; TMB, tumor mutational burden; TP53, tumor protein p53; VHL, Von Hippel-Lindau tumor suppressor | | | | |

Supplementary Table S3. Inter-reader agreements of imaging features

| **Radiologic features** | **ICC or kappa-statistics (95% CI)** |
| --- | --- |
| Attenuation score* | 0.599 (0.366-0.756) |
| Tumor heterogeneity score | 0.456 (0.255-0.556) |
| Multi-cystic appearance | 0.503 (0.169-0.837) |
| Non-smooth margin | 0.566 (0.441-0.692) |
| Growth pattern | 0.597 (0.474-0.720) |
| Nodular enhancement | 0.571 (0.431-0.709) |
| Necrosis | 0.598 (0.389-0.714) |
| Calcification | 0.621 (0.491-0.752) |
| Renal sinus extension | 0.586 (0.458-0.712) |
| Renal vein invasion | 0.739 (0.613-0.864) |
| Renal vein tumor thrombosis | 0.798 (0.691-0.907) |
| Collecting duct invasion | 0.684 (0.549-0.839) |
| Intratumoral vessel | 0.562 (0.448-0.675) |
| CI, confidence interval; ICC, intraclass correlation coefficient  Since the attenuation score is a continuous variable, inter-reader agreement was assessed using ICC. Inter-reader agreements of all other imaging features were assessed using kappa-statistics. | |

Supplementary Table S4. Statistically significant associations (p<0.05) between imaging features and genomic alterations.

| **Radiologic features** |  | **NSM or CNV (+)** | **NSM or CNV (-)** | **p-value** |
| --- | --- | --- | --- | --- |
|  |  | PBRM1 (+) | PBRM1 (-) |  |
| Attenuation score |  | 0.57 (0.43-0.83) | 0.50 (0.36-0.69) | 0.017 |
|  |  | MTOR (+) | MTOR (-) |  |
| Multi-cystic appearance | (+) | 2 (33.3%) | 5 (3.2%) | 0.022 |
|  | (-) | 4 (66.7%) | 153 (96.8%) |  |
|  |  | 3p loss or gain (+) | 3p loss or gain (-) |  |
| Nodular enhancement | (+) | 32 (88.9%) | 86 (67.2%) | 0.011 |
|  | (-) | 4 (11.1%) | 42 (32.8%) |  |
|  |  | 7q loss or gain (+) | 7q loss or gain (-) |  |
| Nodular enhancement | (+) | 17 (94.4%) | 101 (69.2%) | 0.025 |
|  | (-) | 1 (5.6%) | 45 (30.8%) |  |
|  |  | VHL (+) | VHL (-) |  |
| Necrosis | (+) | 116 (91.3%) | 27 (73.0%) | 0.008 |
|  | (-) | 11 (8.7%) | 10 (27.0%) |  |
|  |  | 5q loss or gain (+) | 5q loss or gain (-) |  |
| Necrosis | (+) | 30 (100%) | 113 (84.3%) | 0.015 |
|  | (-) | 0 (0%) | 21 (15.7%) |  |
|  |  | 8q gain (+) | 8q gain (-) |  |
| Necrosis | (+) | 6 (60.0%) | 137 (89.0%) | 0.025 |
|  | (-) | 4 (40.0%) | 17 (11.0%) |  |
|  |  | 7q loss or gain (+) | 7q loss or gain (-) |  |
| Renal vein invasion | (+) | 10 (55.6%) | 30 (20.5%) | 0.003 |
|  | (-) | 8 (44.4%) | 116 (79.5%) |  |
|  |  | 7q loss or gain (+) | 7q loss or gain (-) |  |
| Renal vein thrombosis | (+) | 8 (44.4%) | 27 (18.5%) | 0.026 |
|  | (-) | 10 (55.6%) | 119 (81.5%) |  |
|  |  | SETD2 (+) | SETD2 (-) |  |
| Collecting duct invasion | (+) | 9 (34.6%) | 20 (14.5%) | 0.029 |
|  | (-) | 17 (65.4%) | 118 (85.5%) |  |
|  |  | PTEN (+) | PTEN (-) |  |
| Intratumoral vessel | (+) | 9 (90.0%) | 81 (52.6%) | 0.023 |
|  | (-) | 1 (10.0%) | 73 (47.4%) |  |
|  |  | 5p loss or gain (+) | 5p loss or gain (-) |  |
| Intratumoral vessel | (+) | 13 (81.2%) | 77 (52.0%) | 0.033 |
|  | (-) | 3 (18.8%) | 71 (48.0%) |  |
|  |  | 5q loss or gain (+) | 5q loss or gain (-) |  |
| Intratumoral vessel | (+) | 23 (76.7%) | 67 (50.0%) | 0.014 |
|  | (-) | 7 (23.3%) | 67 (50.0%) |  |
|  |  | 3p loss or gain (+) | 3p loss or gain (-) |  |
| Distant metastasis | (+) | 6 (16.7%) | 48 (37.5%) | 0.031 |
|  | (-) | 30 (83.3%) | 80 (62.5%) |  |
|  |  | 8q gain (-) | 8q gain (-) |  |
| Distant metastasis | (+) | 7 (70.0%) | 47 (30.5%) | 0.015 |
|  | (-) | 3 (30.0%) | 107 (69.5%) |  |
|  |  | 8q gain (-) | 8q gain (-) |  |
| Tumor size (cm) | (+) | 3.70 (3.00-5.65) | 7.00 (4.40-9.80) | 0.007 |
| Data is presented as either number (percentage) or median (25^th^-75^th^ percentile).  Abbreviations: CNV, copy number variation; MTOR, mechanistic target of rapamycin kinase; NSM, nucleotide substitution mutation; PBRM1, Polybromo 1; PTEN, phosphatase and tensin homolog; VHL, SED2, SET domain containing 2; Von Hippel-Lindau tumor suppressor | | | | |

Supplementary Table S5. Genomic profile of cluster C1 and C2 in training- and test-sets.

| **Variable** | **Data** | **Training-set (n=82, 50%)** | | **p-value** | **Data** | **Test-set (n=82, 50%)** | | **p-value** |
| --- | --- | --- | --- | --- | --- | --- | --- | --- |
|  |  | **Cluster 1, C1 (n=52, 63%)** | **Cluster 2, C2 (n=30, 37%)** |  |  | **Cluster 1, C1 (n=54, 66%)** | **Cluster 2, C2 (n=28, 34%)** |  |
| **Nucleotide substitution mutations** | 82 |  |  |  | 82 |  |  |  |
| VHL |  | 40 (76.9) | 22 (73.3) | 0.792 |  | 41 (75.9) | 24 (85.7) | 0.395 |
| EGFR splice |  | 36 (69.2) | 19 (63.3) | 0.631 |  | 38 (70.4) | 18 (64.3) | 0.622 |
| PBRM1 |  | 15 (28.8) | 14 (46.7) | 0.150 |  | 17 (31.5) | 7 (25.0) | 0.615 |
| SETD2 |  | 9 (17.3) | 5 (16.7) | 0.999 |  | 8 (14.8) | 4 (14.3) | 0.999 |
| BAP1 |  | 4 (7.7) | 5 (16.7) | 0.276 |  | 9 (16.7) | 3 (10.7) | 0.743 |
| TP53 |  | 3 (5.8) | 4 (13.3) | 0.253 |  | 5 (9.3) | 3 (10.7) | 0.999 |
| KDM5C |  | 2 (3.8) | 4 (13.3) | 0.185 |  | 4 (7.4) | 2 (7.1) | 0.999 |
| TERT |  | 3 (5.8) | 2 (6.7) | 0.999 |  | 5 (9.3) | 2 (7.1) | 0.999 |
| MTOR |  | 1 (1.9) | 1 (3.3) | 0.999 |  | 4 (7.4) | 0 (0) | 0.294 |
| PTEN |  | 4 (7.7) | 0 (0) | 0.291 |  | 4 (7.4) | 2 (7.1) | 0.999 |
| **Copy-number variations** | 82 |  |  |  | 82 |  |  |  |
| 1q gain |  | 5 (9.6) | 0 (0) | 0.153 |  | 3 (5.6) | 2 (7.1) | 0.999 |
| 3p loss |  | 13 (25.0) | 6 (20.0) | 0.787 |  | 12 (22.2) | 5 (17.9) | 0.777 |
| 5p gain |  | 6 (11.5) | 1 (3.3) | 0.414 |  | 8 (14.8) | 1 (3.6) | 0.156 |
| 5q gain |  | 15 (28.8) | 4 (13.3) | 0.173 |  | 9 (16.7) | 2 (7.1) | 0.316 |
| 7p gain |  | 8 (15.4) | 2 (6.7) | 0.312 |  | 6 (11.1) | 4 (14.3) | 0.729 |
| 7q gain |  | 9 (17.3) | 0 (0) | 0.023 |  | 5 (9.3) | 4 (14.3) | 0.483 |
| 8p loss |  | 7 (13.5) | 2 (6.7) | 0.475 |  | 4 (7.4) | 2 (7.1) | 0.999 |
| 8q gain |  | 0 (0) | 2 (6.7) | 0.131 |  | 4 (7.4) | 4 (14.3) | 0.435 |
| 8q loss |  | 1 (1.9) | 0 (0) | 0.999 |  | 2 (3.7) | 0 (0) | 0.545 |
| 14q gain |  | 1 (1.9) | 0 (0) | 0.999 |  | 0 (0) | 0 (0) | NA |
| 14q loss |  | 2 (3.8) | 2 (6.7) | 0.621 |  | 3 (5.6) | 3 (10.7) | 0.406 |
| 20p gain |  | 3 (5.8) | 0 (0) | 0.295 |  | 3 (5.6) | 1 (3.6) | 0.999 |
| 20p loss |  | 0 (0) | 0 (0) | NA |  | 1 (1.9) | 0 (0) | 0.999 |
| 20q gain |  | 4 (7.7) | 1 (3.3) | 0.648 |  | 4 (7.4) | 1 (3.6) | 0.656 |
| TMB (mut/MB) | 82 | 5.5 (3.9, 7.1) | 5.5 (4.1, 7.6) | 0.742 | 82 | 5.5 (3.9, 6.3) | 5.9 (3.9, 7.9) | 0.210 |
| MSI (%) | 82 | 2.6 (1.9, 3.4) | 2.0 (1.7, 3.4) | 0.238 | 82 | 2.4 (1.6, 3.2) | 2.5 (1.4, 3.3) | 0.751 |
| Data is presented as median (25^th^ – 75^th^ percentile) or number (percentage).  Abbreviations: BAP1, BRCA21 associated protein; EGFR splice, epidermal growth factor receptor slice variant; KDM5C, lysine demethylase 5C; MTOR, mechanistic target of rapamycin; MSI, microsatellite instability; PBRM1, polybromo 1; PTEN, phosphatase and tensin homolog; SETD2, SET domain containing 2; TERT, telomerase reverse transcriptase; TMB, tumor mutational burden; TP53, tumor protein p53; VHL, Von Hippel-Lindau tumor suppressor | | | | | | | | |

Supplementary Table S6. Univariate and multivariate logistic regression analysis for predicting cluster C1.

|  | Univariate analysis | | Multivariate analysis | | |
| --- | --- | --- | --- | --- | --- |
| Variables | Odds ratio  (95% CI) | p-value | Odds ratio  (95% CI) | p-value | VIF |
| Age (years) | 1.00 (0.97 - 1.03) | 0.898 |  |  |  |
| Sex (men) | 0.85 (0.39 - 1.78) | 0.663 |  |  |  |
| Tumor size (cm) | 2.11 (1.72 - 2.73) | <0.001 | 0.91 (0.32-3.20) | 0.810 | 2.435 |
| Attenuation score | 0.30 (0.08 - 1.03) | 0.062 |  |  |  |
| Heterogeneity score |  |  |  |  |  |
| 1~3 | (ref.) |  | (ref.) |  |  |
| 4~5 | 8.55 (4.70-17.46) | <0.001 | 86.73 (7.93-4925.01) | **<0.001** | 3.593 |
| Non-smooth margin | 26.87 (11.57 - 69.75) | <0.001 | 80.91 (5.00-15379.64) | **0.006** | 2.229 |
| Growth pattern |  |  |  |  |  |
| Endophytic or <50% exophytic | (ref.) |  | (ref.) |  |  |
| ≥50% exophytic | 35.56 (14.89 – 94.46) | <0.001 | 185.27 (8.70-164875.37) | **<0.001** | 2.183 |
| Nodular enhancement | 9.44 (4.43 - 21.21) | <0.001 | 1.08 (0.01-30.05) | 0.955 | 1.742 |
| Necrosis (for solid tumor) | 20.64 (6.18-106.43) | <0.001 | 0.01 (0.00-154716.81) | 0.161 | 1.335 |
| Calcification | 5.58 (2.06 - 19.58) | 0.002 | 0.73 (0.00-172.58) | 0.895 | 2.501 |
| Renal sinus extension | 19.65 (8.77 - 48.39) | <0.001 | 50.32 (1.44-173311.56) | **0.026** | 3.311 |
| Renal vein invasion | 35.6 (14.89 – 94.46) | 0.001 | 0.04 (0.00-3335.33) | 0.403 | 3.141 |
| Renal vein tumor thrombosis | 18.24 (2.61 – 165.52) | 0.001 | (-) | (-) | 13.94 |
| Collecting duct invasion | 9.57 (2.72 - 60.85) | 0.003 | 7.84 (0.01 – 4771.82) | 0.457 | 2.717 |
| Intratumoral vessel | 9.19 (4.45 - 20.10) | <0.001 | 2.29 (0.65 – 8.44) | 0.196 | 2.471 |
| cT stage |  |  |  |  |  |
| 1~2 | (ref.) |  | (ref.) |  |  |
| 3~4 | 6.35 (3.17 - 13.25) | <0.001 | 1.34 (0.19-9.45) | 0.767 | 2.505 |
| cN1 | 2.35 (1.04 - 5.89) | 0.051 |  |  |  |
| cM1 | 2.18 (1.07 - 4.66) | 0.036 | 1.42 (0.17-12.74) | 0.743 | 1.945 |
| VIF, variance inflation factor  Renal vein tumor thrombosis was not analyzed in the multivariate analysis due to multicollinearity (VIF=13.94). | | | | | |


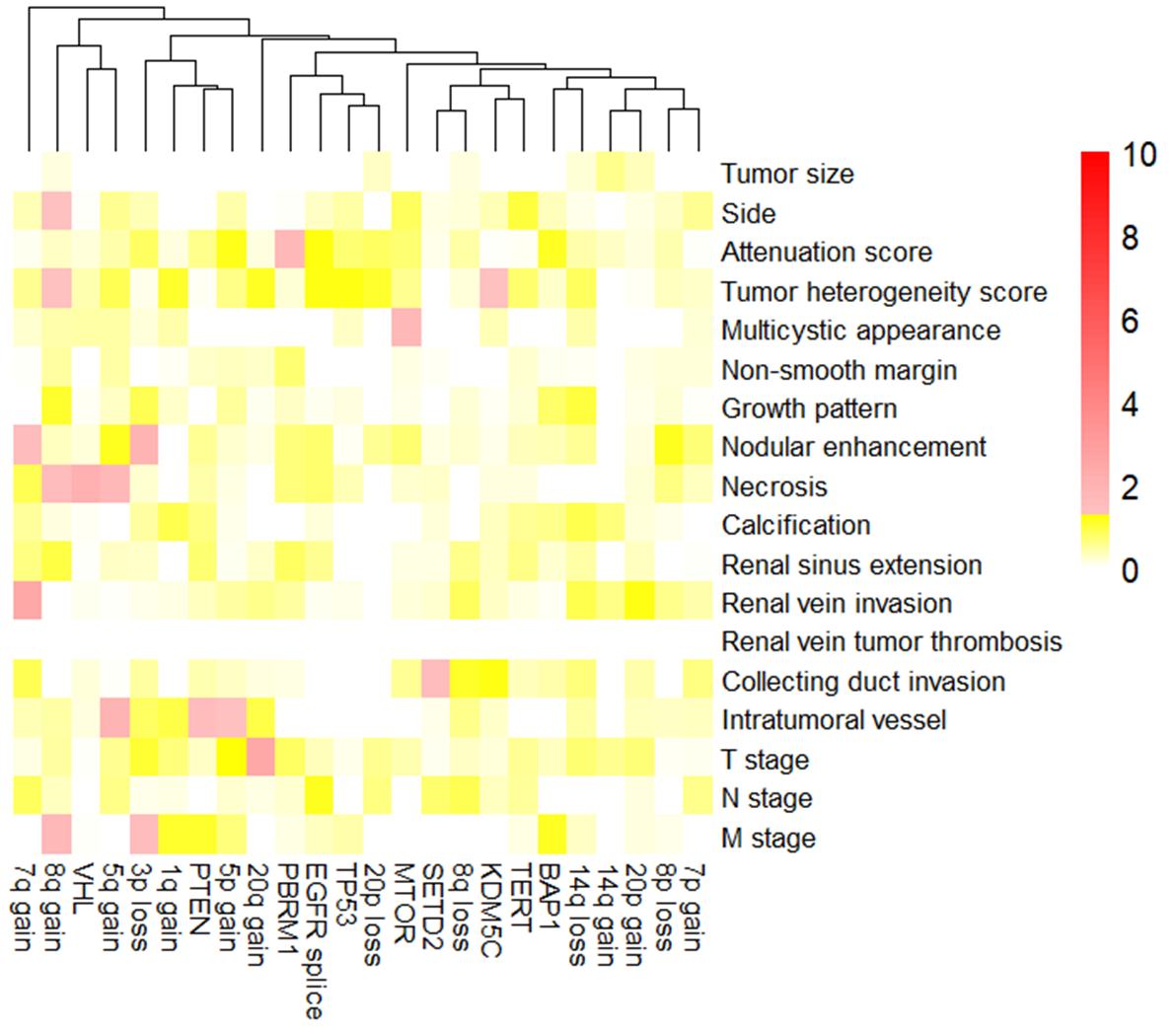


Supplementary Fig S1. Heatmap showing the associations between imaging features and genomic alterations in ccRCC patients. Color scale: 0 (white), no association; 10 (red): -log10(p) = 10, highly significant (p<0.001).


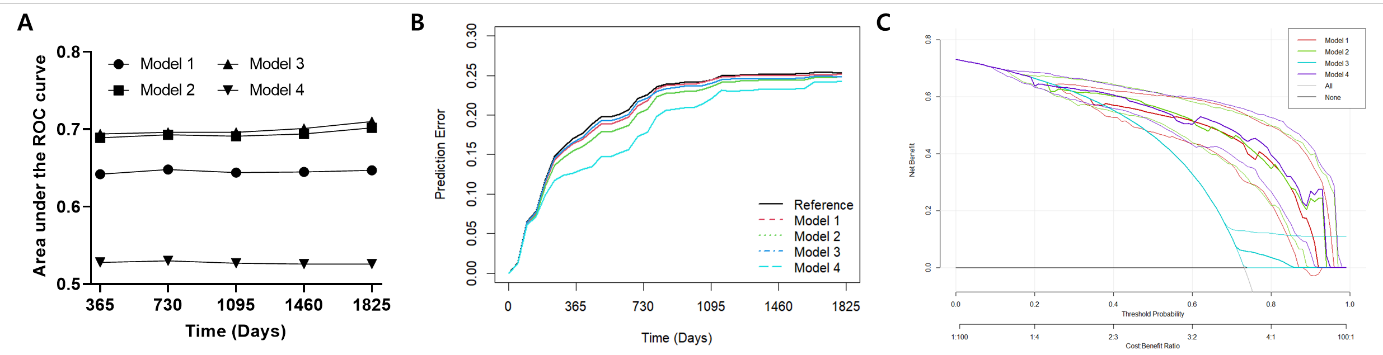


Supplementary Fig. S2. Time-dependent ROC curves, prediction error curves and decision-curves for models 1-4 in the test-set. ROC, receiver operating characteristic curve.

**References**

1 Cha YJ, Lee C, Joo B, Kim KA, Lee CK, Shim HS (2023) Clinicopathological Characteristics of NRG1 Fusion-Positive Solid Tumors in Korean Patients. Cancer Res Treat 55:1087-1095

2 Li MM, Datto M, Duncavage EJ et al (2017) Standards and Guidelines for the Interpretation and Reporting of Sequence Variants in Cancer: A Joint Consensus Recommendation of the Association for Molecular Pathology, American Society of Clinical Oncology, and College of American Pathologists. J Mol Diagn 19:4-23

3 Schneider VA, Graves-Lindsay T, Howe K et al (2017) Evaluation of GRCh38 and de novo haploid genome assemblies demonstrates the enduring quality of the reference assembly. Genome Res 27:849-864

4 Karczewski KJ, Francioli LC, Tiao G et al (2020) The mutational constraint spectrum quantified from variation in 141,456 humans. Nature 581:434-443

5 Richards S, Aziz N, Bale S et al (2015) Standards and guidelines for the interpretation of sequence variants: a joint consensus recommendation of the American College of Medical Genetics and Genomics and the Association for Molecular Pathology. Genet Med 17:405-424

6 Talevich E, Shain AH, Botton T, Bastian BC (2016) CNVkit: Genome-Wide Copy Number Detection and Visualization from Targeted DNA Sequencing. PLoS Comput Biol 12:e1004873

7 Haas BJ, Dobin A, Li B, Stransky N, Pochet N, Regev A (2019) Accuracy assessment of fusion transcript detection via read-mapping and de novo fusion transcript assembly-based methods. Genome Biol 20:213

8 Mayakonda A, Lin DC, Assenov Y, Plass C, Koeffler HP (2018) Maftools: efficient and comprehensive analysis of somatic variants in cancer. Genome Res 28:1747-1756

9 Smyth EC, Verheij M, Allum W, Cunningham D, Cervantes A, Arnold D (2016) Gastric cancer: ESMO Clinical Practice Guidelines for diagnosis, treatment and follow-up. Ann Oncol 27:v38-v49
